# The gut neuroepithelial unit as a neurodegeneration-relevant interface in the gut–brain axis

**DOI:** 10.1186/s40035-026-00582-0

**Published:** 2026-09-01

**Authors:** Siqi Guo, Rui Guo, Qingyu Zhang, Liyuan Fu, Huaxu Zhu, Qichun Zhang

**Affiliations:** 1 Jiangsu Key Laboratory for Pharmacology and Safety Evaluation of Chinese Materia Medica, School of Pharmacy, Nanjing University of Chinese Medicine, Nanjing, 210023 China; 2 Jiangsu Key Laboratory for High Technology Research of TCM Formulae, Nanjing, University of Chinese Medicine, Nanjing, 210023 China; 3 School of Medicine, Nanjing University of Chinese Medicine, Nanjing, 210023 China; 4 School of Chinese Medicine, Nanjing University of Chinese Medicine, Nanjing, 210023 China; 5 Jiangsu Collaborative Innovation Center of Chinese Medicinal Resources Industrialization, Nanjing University of Chinese Medicine, Nanjing, 210023 China

**Keywords:** Gut neuroepithelial unit, Signal encoding, Microbiota-gut-brain axis, Neuroimmune communication, Translational pharmacology

## Abstract

Neurodegenerative diseases are increasingly linked to abnormalities in the gut–brain axis, yet the local intestinal interface at which luminal and mucosal perturbations are related with the central nervous system remains poorly defined. The gut neuroepithelial unit (GNU) is proposed as a localized mucosal signalling interface composed of sensory epithelial cells, enteric neurons, glia and adjacent immune-stromal elements that detect, encode and route intestinal information into neural, endocrine and immune outputs. This framework shifts the intestine from being a diffuse upstream modifier of brain pathology to a mesoscopic unit through which microbial products, barrier dysfunction, inflammatory cues and metabolic signals are transformed into disease-relevant gut-to-brain communication. Particularly, in Parkinson’s disease and Alzheimer’s disease, the GNU may function as a conditional interface that amplifies, filters or biases peripheral signals before they engage central circuits. The GNU therefore provides a tractable framework for mechanistic dissection, translational stratification and peripheral therapeutic targeting in neurodegeneration.

## Introduction

The gut–brain axis is increasingly recognized as a bidirectional communication network linking intestinal physiology to central nervous system (CNS) function. Yet the local interface through which intestinal perturbations are signaled to the CNS remains poorly defined. Recent reviews have linked intestinal dysfunction, dysbiosis, microbial metabolites, barrier disruption, autonomic signalling and neuroinflammation to neurodegenerative diseases, including Alzheimer’s disease (AD), Parkinson’s disease (PD) and Huntington’s disease (HD) [1–6]. This suggests that the intestinal mucosa functions not merely as a boundary, but as an active site at which local disturbances may have systemic significance. In this context, the intestinal epithelium serves as a selective transductive surface that senses luminal and tissue-derived cues and converts them into biologically effective signals [7, 8]. Existing frameworks capture important aspects of intestinal organization, but remain functionally incomplete. For example, the neuronal–glial–epithelial unit framework emphasizes barrier regulation and mucosal homeostasis, whereas the neuroepithelial circuit models prioritize temporal precision and vagal engagement. There is a need for an integrated framework capable of accommodating the convergence of microbe-epithelium-nerve communication. The novelty of the present review is to shift this discussion from pathway-level gut-brain association to a defined mucosal encoding interface at which luminal and tissue-derived cues are selected, formatted and routed into brain-relevant outputs.

Here, the gut neuroepithelial unit (GNU) is proposed as the minimal functional and anatomical module for gut-to-brain transduction. The GNU is defined as a spatiotemporally coordinated ensemble of sensory epithelial cells, enteric neurons, glia and adjacent immune-stromal elements. At this scale, rapid neurotransmission, secretory responsiveness and immune surveillance are integrated within a single mucosal interface, providing an anatomically tractable framework for mechanistic investigation. The GNU also offers a conceptual basis for understanding how localized mucosal perturbations are related to the CNS. In this way, it reframes gut-originating pathology as dysfunction within a localized multicellular interface and facilitates higher-resolution targeting along the gut–brain axis.

## The GNU in comparative physiology

Across organ systems, localized sensing is commonly coupled to spatially restricted effector pathways, despite the different cellular compositions and physiological outcomes. In the airway, pulmonary neuroepithelial bodies (NEBs) function as a polymodal sensory structure that couples local stimuli directly to afferent pathways [9, 10]. A comparable organizational logic is found in the kidney, where the macula densa serves as a specialized epithelial sensor of luminal NaCl and metabolic cues, converting these inputs into paracrine control of renin release and glomerular haemodynamics [11–13]. Such examples suggest that localized sensing units represent a recurring physiological solution across diverse tissues. Although constituent cells vary according to tissue architecture and functional demand, they share a common principle: local detection is linked to spatially constrained output pathways.

A feature of the intestine is the requirement to process chemically diverse, microbially dense and temporally fluctuating cues within a tissue in which epithelial, neural, glial, immune and stromal elements are already in close proximity [14]. Studies of enteroendocrine cells have established the intestinal epithelium as an active sensory interface within the gut–brain axis [7, 14, 15]. Direct experimental evidence is the identification of neuropod cells, a specialized enteroendocrine subset that forms synapse-like junctions with vagal afferents and enables millisecond-scale transmission of luminal information [16]. Subsequent functional analyses have shown that these sensory elements can influence physiological outputs, ranging from nutrient preference to sympathetic circuits that shape the systemic metabolism and cognitive state [17–19]. Together, these findings indicate that the intestinal epithelium contains specialized sensory elements capable of rapid and high-fidelity neural communication.

**Fig. 1:**
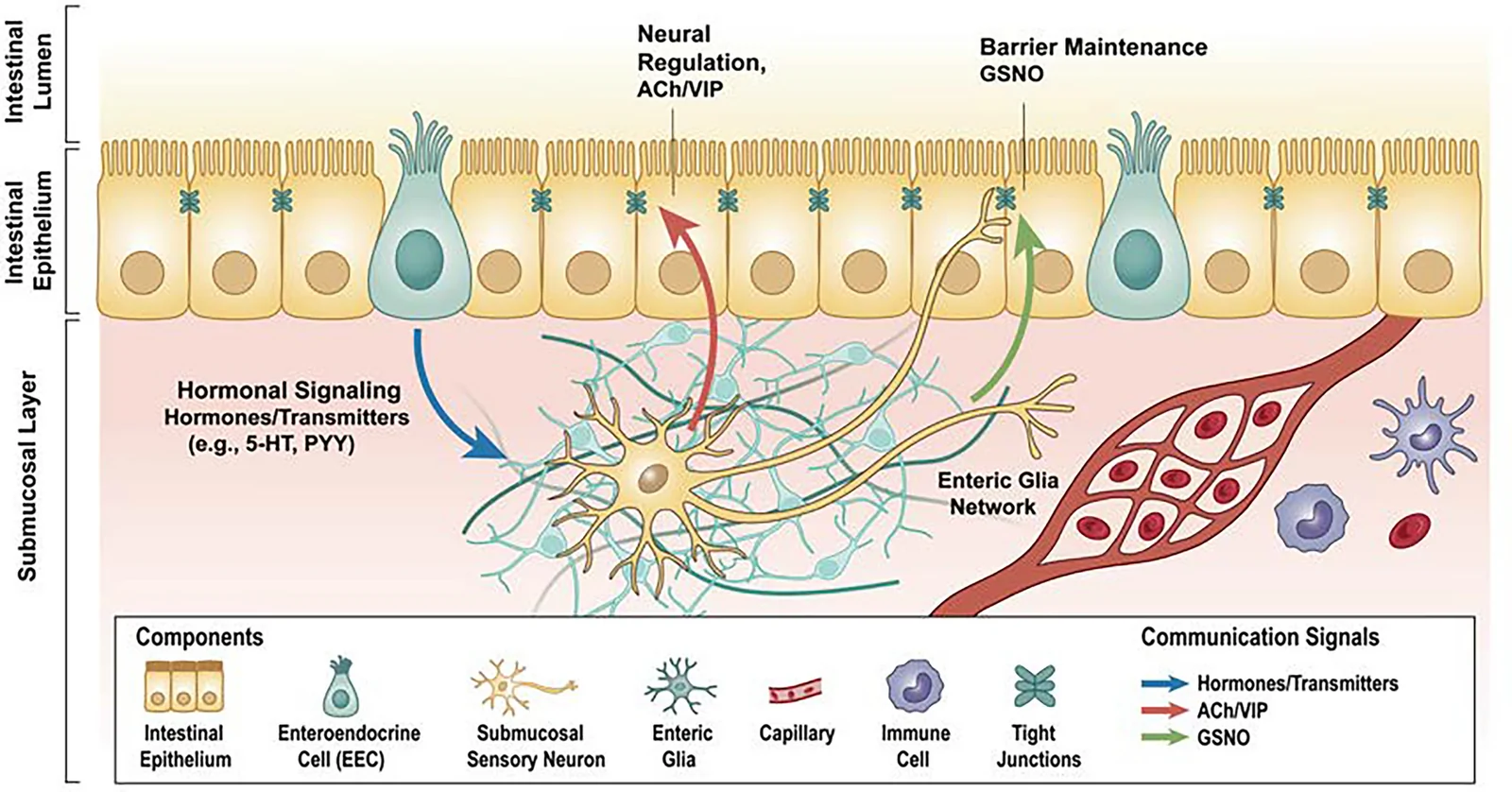
Cellular composition and local signalling architecture of the GNU. The GNU is shown as a localized mucosal microdomain comprising sensory epithelial cells, submucosal sensory neurons, enteric glia, immune cells and adjacent microvasculature. Enteroendocrine cells span the intestinal epithelium and relay luminal information to nearby neural elements through hormones and neurotransmitters, including 5-hydroxytryptamine (5-HT) and peptide YY (PYY). Submucosal sensory neurons provide a route for neuroepithelial signal relay, whereas reciprocal epithelial regulation is mediated by neural transmitters such as acetylcholine and vasoactive intestinal peptide. Enteric glia form a local regulatory network that supports epithelial integrity and barrier maintenance, including through mediators such as S-nitrosoglutathione. Together, these elements define the GNU as a spatially restricted interface in which epithelial sensing, neural relay, glial modulation and immune surveillance are anatomically coupled

These observations further suggest that the intestinal sensing cannot be reduced to epithelial-neuronal coupling alone, but instead requires a multicellular mucosal framework in which glial regulation, immune surveillance and stromal context shape the signal transmission [20–23]. Mucosal immune cells act as dynamic sentinels that continuously monitor barrier integrity and microbial cues through reciprocal exchange with neural and epithelial compartments [24–26]. These intersecting pathways converge within a spatially confined yet functionally integrated mucosal microdomain. By integrating epithelial transduction, neuronal relay, glial buffering and immune-stromal modulation, the GNU translates luminal perturbations into coordinated local and systemic outcomes. This integrative perspective leads to a more specific need: to define the cellular architecture of the GNU with greater anatomical and functional precision. It is therefore essential to resolve the spatiotemporal principles governing GNU assembly and its orchestration within the intestinal mucosa (Fig. 1).

## Cellular organization of the GNU

The epithelial interface of the GNU comprises specialized sensory populations. Single-cell transcriptomic studies have revealed substantial regional diversity of intestinal epithelial populations, with distinct secretory and chemosensory subtypes distributed along the gastrointestinal tract [27, 28]. Enteroendocrine cells constitute the primary luminal detection layer, sensing metabolic cues and converting them into hormonal or neural signals [28]. Enterochromaffin cells add a mechanochemical dimension to this circuitry by coupling physical and chemical stimuli to serotonin release and subsequent sensory nerve activation [29, 30]. Tuft cells further broaden this sensory repertoire by detecting parasite-derived factors and initiating type 2 immune programs through interleukin (IL)-25-dependent pathways [31–33]. The epithelial arm of the GNU therefore functions as a stratified sensory layer rather than as a single cellular entity.

Although neuropod cells provide one of the most direct structural mechanisms for rapid epithelial–neural communication, the neuronal component of the GNU extends beyond this specialized relay and includes diverse enteric neural circuits that integrate epithelial, glial, stromal, and immune signals locally [27]. This supports precise nutrient discrimination and contributes to downstream behavioural modulation [32]. Beyond these direct relays, the enteric nervous system (ENS) comprises multiple neuron classes and has connections among all classes, which integrate epithelial, stromal and immune sensing [34, 35]. Within the GNU, neurons function not only as signal recipients but also as active processors of mucosal information. Enteric glia form a regulatory layer with state-dependent functions. Under basal conditions, they stabilize local physiology and support motility, whereas during inflammation or stress, they can reshape barrier properties, neuronal viability and neuroimmune communication [36–38]. This functional plasticity suggests that the GNU can shift from a homeostatic to a maladaptive state depending on the microenvironment.

Within the GNU, immune cells are not merely responders, but they also participate in information processing, translating signals arising from barrier disruption, microbial exposure and neural activity into context-dependent immune responses, including inflammatory or reparative states [23]. Macrophage-neuron crosstalk regulates intestinal motility and tissue adaptation, whereas neuropeptide-mediated regulation of functions of innate lymphoid cells is a mechanism linking neural activity to epithelial repair [39–41]. Under inflammatory conditions, glia and immune cells are also engaged in bidirectional injury propagation [38].

A minimal GNU therefore comprises a sensory epithelial cell and its immediate neural, glial, immune and stromal partners, which cooperate for local signal integration and transmission. Coupling of these units through enteric circuits and paracrine mediators can convert luminal signals into coordinated reflexes and immune programmes. In this way, the GNU provides a structural basis for translating gut-derived information into brain reactivity.

## Comparison of GNU with other sensing systems

Unlike the diffusional epithelial-neural communication seen in many barrier tissues, the GNU contains specialized neuroepithelial contacts that support faster and more spatially restricted transmission [45]. Intestinal epithelial sensors form synapse-like junctions with afferent neurons, enabling rapid and spatially precise signaling [7, 46]. Under physiological conditions, the GNU responds to subtle changes in luminal nutrients and microbial metabolites [23, 42]. In this respect, it is more spatially constrained and functionally integrated than the diffusional signaling typical of many epithelial-immune interfaces [43, 44].

A related organizational principle can be seen in NEBs, which respond to hypoxia and pH changes through the release of serotonin and neuropeptides [9, 10]. Both GNU and NEB systems rely on localized sensing, but they differ in cellular diversity and in the range of stimuli they process [9, 47]. NEBs comprise a relatively restricted and homogeneous cell population, whereas the GNU incorporates multiple epithelial lineages, including enteroendocrine, enterochromaffin and tuft cells [42, 46, 48, 49], in addition to enteric glia and immune cells as integral components. Furthermore, the GNU must process a broader range of inputs, including microbial products and inflammatory signals, within the same local interface [42, 48, 49].

The macula densa is a specialized epithelial plaque in the distal nephron. It senses luminal NaCl and metabolic cues and converts these inputs into paracrine regulation of renin release and glomerular haemodynamics [11, 12]. Like the GNU, it couples local sensing to organ-level homeostasis [11]. However, it lacks the rapid synaptic communication characteristic of the intestinal interface [7, 46], and its cellular composition is comparatively fixed. Unlike relatively fixed sensory structures, the GNU remains functionally plastic because the incorporation of enteric glial and immune components allows dynamic adjustment of epithelial–neural communication according to local physiological and inflammatory states [43, 44]. This heterogeneity of components in GNU probably reflects the need to process variant signals, including nutrient, microbial, mechanical and inflammatory, within a single mucosal compartment [12].

Taste buds and the carotid body are further examples of localized receptor-effector organization. In both systems, specialized receptor cells activate sensory afferents through transmitter release [50, 51]. A difference is that the receptor-like epithelial sensing in GNU is embedded within a tissue that must simultaneously manage microbial surveillance, barrier integrity and local immune regulation [23, 42–44].

## Signalling architecture of the GNU

Once the cellular architecture of the GNU is established, the next question is how its components communicate in temporal and spatial scales [42]. The communication includes neural, endocrine, immune and microbial-metabolic pathways, which operate in parallel but are functionally interconnected [46]. Among them, neural signaling provides the highest temporal precision and is therefore central to the rapid stimulus classification [7]. Enterochromaffin cells act as primary chemosensors and release serotonin in response to luminal stimuli, activating nearby sensory neurons [44]. Neuropod cells form synapse-like contacts with vagal afferents and transmit nutrient-related information by secreting glutamate on a millisecond timescale [52]. This epithelial-neuronal coupling enables rapid, directional and high-fidelity signal transfer [53]. Vagal afferent fibres, which constitute the majority of fibres within the vagus nerve, are anatomically positioned to transmit these signals to the brainstem [54]. In addition to long-range transmission, intrinsic primary afferent neurons can synapse with interneurons within the ENS, generating local reflexes [55].

Endocrine and humoral pathways extend GNU signaling across longer temporal and spatial ranges, allowing local epithelial detection to influence distant metabolic and central targets [42]. Enteroendocrine cells release peptide hormones such as glucagon-like peptide 1 (GLP-1), PYY and cholecystokinin (CCK) in response to nutrient stimulation [23]. GLP-1 functions both as a circulating incretin hormone and as a local modulator of vagal afferent activity [56]. Similarly, PYY contributes to appetite suppression through both endocrine and neural routes [57]. These hormones act via the hypothalamus to modulate appetite-related circuits, while also modulating peripheral metabolic organs [56]. This dual function illustrates how endocrine and neural pathways converge within the GNU, where locally generated hormonal signals can modulate sensory circuits while also exerting systemic metabolic effects [46]. More broadly, gut–brain metabolic communication relies on coordinated interactions between neural feedback pathways and circulating endocrine signals that together regulate energy homeostasis [25].

The microbial-metabolic pathway enables the GNU to detect and integrate microbiota-derived cues directly at the mucosal interface [58]. Short-chain fatty acids (SCFAs) generated by bacterial fermentation act on enteroendocrine cells through G-protein-coupled receptors, stimulating GLP-1 secretion [27]. Propionate similarly induces PYY release, linking microbial metabolism to appetite regulation [59]. Tuft cells add a further layer by detecting luminal metabolites and initiating type 2 immune responses through IL-25 signaling [60].

The immune and inflammatory components of the GNU enable continuous monitoring of barrier integrity and host defence status by integrating microbial, epithelial and tissue-derived signals [43]. Intestinal macrophages interact directly with enteric neurons to regulate gut motility and tissue homeostasis [62]. Neural inputs, including sympathetic signaling, can in turn reprogramme macrophage phenotypes and influence inflammatory responses [63]. Enteric glia sense inflammatory cues and modulate neuronal activity [64]. Under pathological conditions, glial activation can contribute to neuronal injury through purinergic and nitric oxide-dependent mechanisms [65]. Cytokines released in the gut may enter the circulation or signal through neural routes to shape the central immune responses [48]. Together, these findings position the GNU as a neuroimmune interface through which barrier perturbation can be translated into both local and systemic consequences [43, 49].

**Fig. 2:**
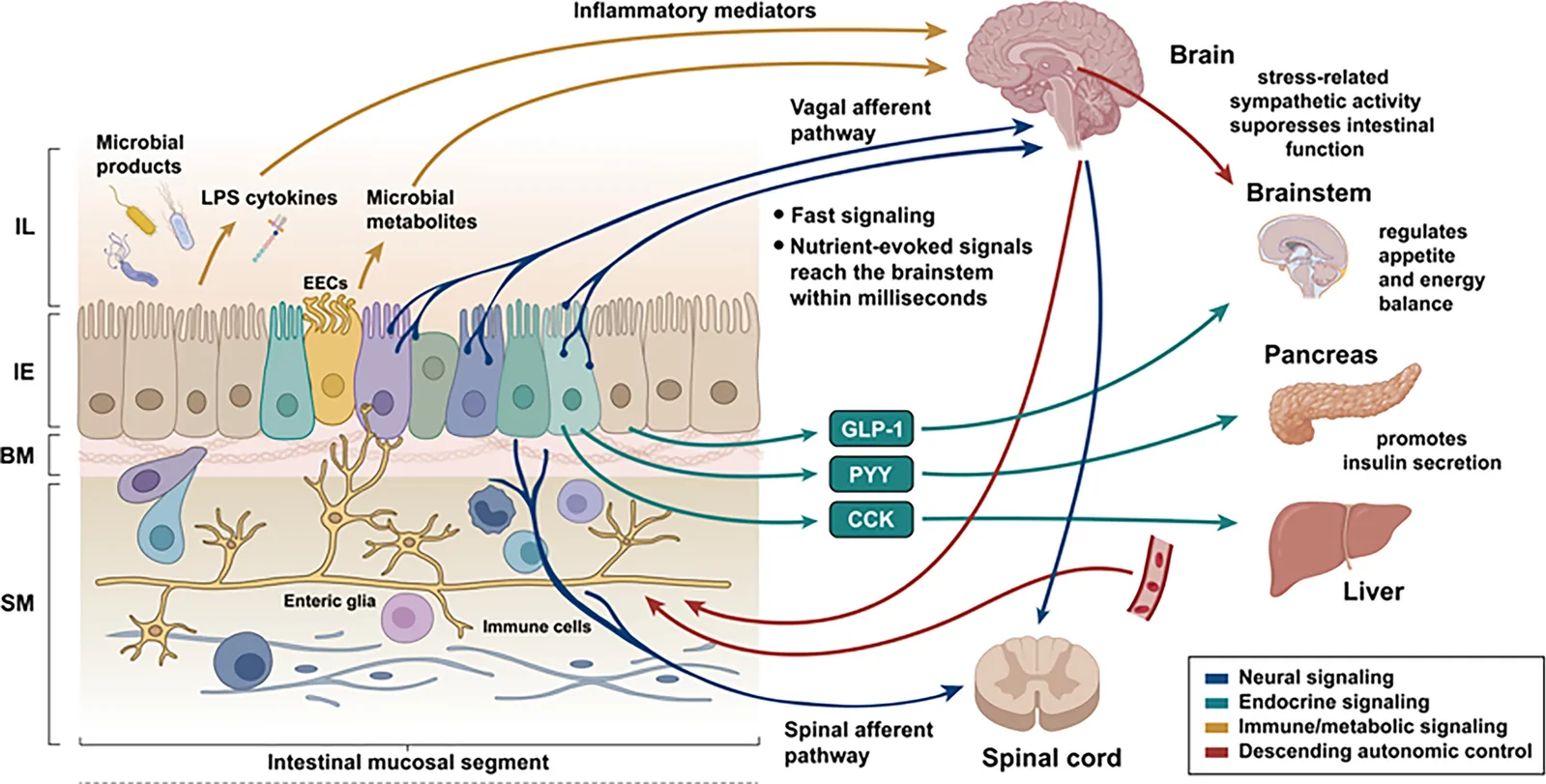
Signal integration and cross-system communication of the GNU. The GNU is a localized mucosal interface through which luminal, microbial, inflammatory and metabolic cues are integrated into neural, endocrine and immune signaling pathways. Fast neural transmission is conveyed through vagal and spinal afferents, allowing nutrient-evoked signals to reach the brainstem, spinal cord and higher central circuits within milliseconds. In parallel, enteroendocrine mediators, including GLP-1, PYY and CCK, engage endocrine routes that influence appetite, energy balance and peripheral metabolic organs, including the pancreas and liver. Microbial products, lipopolysaccharide (LPS), cytokines and related inflammatory mediators provide an additional immune-metabolic channel through which mucosal perturbation can influence central inflammatory tone. Descending autonomic control from the brain back to the intestinal interface is also depicted, emphasizing that the GNU functions both as an ascending sensory gateway and as a target of central regulation

The signaling modalities described above are closely interconnected rather than functionally independent [46]. Microbial metabolites can trigger endocrine responses [27], the endocrine signals can modulate neural circuits [56], and immune mediators can alter neuronal excitability and glial function [64]. Neural responses can reshape immune responses through vagal and sympathetic pathways [49]. The GNU is therefore better understood not as a collection of parallel routes, but as an integrated signaling interface. This raises the next question: how are these different modalities organized into structured signal encoding [46, 61] (Fig. 2).

## Encoding principles of the GNU

The intestinal mucosa does not relay luminal events as diffuse chemical exposure. Instead, signals are classified at the point of entry and assigned a transmitter format, a spatial route and a temporal scale before they engage enteric, vagal or spinal circuits [16, 44, 66, 67]. The key question is therefore not simply which mediators are released in the gut, but how epithelial and neuroepithelial elements transform distinct luminal and tissue-derived cues into structured neural information. The first layer of this encoding process resides in the epithelium. Sensory epithelial cells recruit distinct receptors and transduction modules for nutrients, bitter compounds, microbial products, inflammatory cues and mechanical perturbations [44, 68, 69]. This organization also determines whether a signal is delivered as a rapid synaptic event, a short-range paracrine relay or a slower endocrine-like output [67, 68, 70]. The GNU therefore functions as a peripheral encoding surface rather than as a passive endocrine sheet.

A clear example is the intestinal sweet sensing. In the duodenum, neuropod-forming enteroendocrine cells distinguish caloric sugars from non-caloric sweeteners and transmit these signals through different neurotransmitters. Glucose sensing through SGLT1 (sodium–glucose cotransporter 1) preferentially drives glutamatergic signaling to vagal neurons, whereas sucralose sensing through sweet taste receptors preferentially engages purinergic transmission [16]. The structural basis of this process is equally important. Neuropods which form direct contacts with afferent neurons, contain ultrastructural features consistent with the dual signaling modes, including clear vesicles for rapid neurotransmission and dense-core vesicles for peptide release [68]. This organization allows both fast neurotransmission and dense-core vesicle release extending the contextual influence [67, 68, 70].

ATP and glutamate appear to be major components of this fast code. In canonical taste pathways, ATP acts through purinergic receptor P2X2 and P2X3 pathways to transfer sensory epithelial information to afferent fibres, and CALHM (calcium homeostasis modulator)-dependent release supports rapid chemosensory transmission [71]. The same pathway is used by intestinal sensor cells to detect non-caloric sweeteners [16]. Glutamate provides the complementary channel for caloric nutrient detection [16, 71]. This suggests that the gut uses a transmitter architecture for rapid sensory discrimination rather than relying solely on hormonal diffusion.

In addition, enterochromaffin cells respond to luminal, microbial and mechanical cues by releasing 5-HT, which influences secretion, motility and afferent excitability [23, 72]. Recent circuit studies using Ca^2+^ imaging further found that different nutrients activate distinct ensembles of neurons through the epithelium, with 5-HT involved in the activation of enteric neurons through mucosal nerve endings [73]. This finding advances the understanding of the role of mucosal serotonin. 5-HT is not merely a universal trigger of gut reflexes; within the GNU, it functions more as an intermediate coding layer that redistributes epithelial information into circuit-specific activity [44, 72, 73].

Peptide mediators encode a slower and more contextual level of information. CCK, GLP-1, GIP (glucose-dependent insulinotropic polypeptide) and PYY are traditionally recognized as metabolic hormones; however, within the GNU they also convey information about nutrient availability, intestinal physiological state and metabolic context by modulating local sensory circuits and downstream neural pathways [67, 70]. The release of these peptides reflects classes of nutrients, the intestinal region, the absorptive stage and the epithelial state [67, 70, 74]. They can act locally on afferent terminals and enteric neurons, while also engaging systemic pathways once they enter the circulation [67, 70, 75]. Their role is not to replace neural transmission, but to assign physiological meaning to rapid epithelial events by linking them to satiety, glycaemic control, absorptive progress and broader metabolic status [67, 70]. For example, luminal lipids and proteins induce release of CCK, which then shapes vagal activity, and controls food intake and glucose handling [70]. This is not a generic satiety effect. It therefore represents a nutrient-specific encoding process in which intestinal signals are transformed into neural information that reflects nutrient identity and physiological context for downstream circuits. PYY operates on a distinct temporal and spatial profile, especially in distal gut feedback, yet it also acts locally within mucosal networks [75, 76]. Fast transmitters identify immediate stimulus class, whereas peptides stabilize and weight that information according to ongoing physiological context.

Paracrine coupling between epithelial transmitter systems adds a further layer of complexity. Incretin-producing cells and enterochromaffin cells interact locally, indicating that peptide and monoamine pathways do not function in an isolated manner [75]. A clear example is the female visceral hypersensitivity. Estrogen signaling upregulates Olfr78 on colonic L cells, increases the acetate-evoked PYY release. The increased PYY acts on neighbouring enterochromaffin cells via NPY1R, thereby enhancing serotonin release and promoting visceral hypersensitivity [76].

Bitter receptor pathways are another dimension of GNU signaling. Bitter taste receptors TAS2Rs in the gut detect luminal compounds with potential defensive relevance, including dietary bitter molecules, microbial products and xenobiotic cues. Their activation engages taste-related intracellular signaling, increases intracellular calcium and promotes mediator release that influences secretion, motility and feeding-related behaviour [69]. In this way, bitter receptor pathways extend GNU function beyond nutrient appraisal by enabling the intestine to classify chemical threats and initiate defensive responses [66, 69].

Spatial organization is central to the functioning of this system. Direct epithelial-neuronal contacts, such as neuropod synapses, support rapid and target-selective transmission [16, 68]. Paracrine signals such as serotonin and peptide mediators diffuse across the mucosal microenvironment and recruit broader networks that include intrinsic primary afferent neurons, secretomotor neurons and extrinsic sensory endings [44, 75]. A single epithelial event can therefore be represented simultaneously in signals that differ in resolution, persistence and circuit destination. Inflammation and tissue injury can further alter GNU signal processing by changing the sensitivity of sensory cells, amplifying or reducing responses to incoming stimuli, and modifying the functional connectivity between epithelial, neural, glial and immune components.

Neuroimmune mediators can modify gut–brain communication by increasing the sensitivity of afferent pathways and altering interactions between epithelial sensory cells and neural circuits [23]. For example, guanylate cyclase 2C-expressing neuropod cells have been implicated in visceral pain regulation, suggesting that sensory epithelial cells can actively shape nociceptive responses rather than simply transmit peripheral information [77]. In addition, ion channels such as NaV1.8 contribute to the transformation of sensitized peripheral stimuli into persistent pain signalling by regulating neuronal excitability [78]. Therefore, under inflammatory or injury conditions, pathological changes do not merely increase the intensity of intestinal signals; they alter how signals are detected, processed and transmitted within the GNU interface [23, 76–78].

These observations support four fundamental principles underlying GNU signal processing. First, receptor expression determines which classes of luminal or tissue-derived stimuli can be detected. Second, the identity of released transmitters and mediators determines the temporal characteristics and mode of communication. Third, the spatial organization of epithelial, neural, glial and immune components determines how information is routed through local and ascending pathways. Fourth, physiological and inflammatory states regulate the sensitivity and magnitude of these responses. Thus, the GNU can be viewed as a peripheral information-processing interface that transforms diverse intestinal signals into structured neural and endocrine outputs before they influence central circuits. Rather than receiving unprocessed intestinal chemical information, the brain receives signals that have already been filtered, integrated and modified by the mucosal environment.

## GNU within the gut–brain axis

Current models of gut–brain axis already support a bidirectional architecture linking microbial metabolites, immune signals, neuroendocrine pathways and autonomic circuits [8, 79, 80]. At the mucosa level of GNU, luminal changes are either encoded as actionable interoceptive information or remain local events [8, 28].

The ascending role of GNU depends on rapid epithelial transduction and an anatomically constrained neural programme that transmits nutrient, microbial and metabolic information into enteric and central circuits [46]. A subset of enteroendocrine cells forms neuropods and establishes direct synaptic communication with vagal neurons [7]. A recent study showed that PYY-labelled colonic neuropod cells detect bacterial flagellin through TLR5 and then release PYY onto NPY2R vagal nodose neurons to suppress feeding [81]. This finding indicates that a microbial pattern can enter the gut–brain axis as a sensory signal before it is expressed as overt inflammation [8]. Endocrine and metabolic signaling follow the same principle. Enteroendocrine cells are key sensors of the gut microbiota and can produce a broad spectrum of hormones to regulate appetite, digestion and glucose homeostasis. Enteroendocrine cell exposure to gut microbiota causes fluctuations in gut hormones such as GLP1, PYY, CCK, and 5-HT, which can act on gastrointestinal nerve fibres and central circuits involved in appetite, stress, and energy allocation [8, 28]. Indigenous bacteria increase host colonic serotonin biosynthesis, and SCFAs further promote enterochromaffin 5-HT production [82, 83].

GNU also plays an important role in descending pathways. Brain-to-gut communication acts through the GNU by resetting epithelial responsiveness, afferent gain, glial state and mucosal immune tone, rather than by acting on the intestine as a uniform target [8, 23, 66, 84]. The vagus nerve is central to this pathway because it contains both afferent and efferent fibres and is closely implicated in gut–brain communication, mood regulation, appetite control and intestinal homeostasis [8, 84]. Vagal and sympathetic nerve activity regulates mucus secretion, absorption, barrier properties and microbial ecology at the intestinal interface [8]. Peripheral immune signals are also involved in this circuitry. A recent study identified a body–brain circuit through which peripheral pro-inflammatory and anti-inflammatory cytokines act on distinct populations of vagal neurons, and the brain in turn regulates peripheral inflammatory responses [85]. Enteric glia are partners of enteric neurons and interact with enteroendocrine cells, epithelial cells, and immune cells to stabilize ENS homeostasis [36, 66].

This bidirectional organization may also create a point of vulnerability under pathological conditions. When GNU signaling is chronically disrupted, altered mucosal states may be transmitted through ascending neural and immune pathways, while descending regulatory signals from the brain may become insufficient to restore local homeostasis. Consequently, barrier dysfunction, microbial imbalance and altered microbial metabolism may have broader physiological consequences because they disrupt the epithelial, neural and immune processes that normally coordinate intestinal sensing and response [1, 8, 36, 79]. Given that the GNU integrates epithelial, neural and immune signalling, persistent dysfunction of this interface may contribute to abnormal gut–brain communication and increase susceptibility to neurological disorders [79, 80, 86]. Importantly, this framework does not suggest that all brain diseases originate from the gut; rather, it identifies the GNU as a potential regulatory interface through which intestinal dysfunction may influence disease vulnerability, progression and phenotypic expression in susceptible individuals [8, 86, 87].

## GNU in CNS diseases

The GNU places epithelial sensing, enteroendocrine transduction, enteric glial modulation, neuroimmune crosstalk and neural relay within the same restricted interface. This is important because luminal changes are selected, amplified or attenuated at the mucosal surface before being transmitted to the brain. In this sense, the GNU functions more as a local decision layer for peripheral signals.

In PD, the GNU participates early in pathogenesis rather than a mere reflection of established central pathology [3, 88, 91]. Gastrointestinal α-synuclein (α-syn) is detectable in patients with PD, and the diagnostic yield is influenced by anatomical site and tissue depth. Upper gastrointestinal tissue performs better than lower gastrointestinal tissue, and longer disease duration predicts greater gastrointestinal α-syn accumulation [86]. These findings have been interpreted within a gut-first framework, in which pathological processes may engage the gastrointestinal tract and vagal circuitry before the emergence of classical motor syndromes [3, 91]. Microbial changes, barrier stress, enteroendocrine signaling, enteric neural dysfunction and mucosal inflammation can converge on the GNU before being transmitted ascendingly through the vagal or autonomic route. This does not imply that PD begins in the gut in every patient. Rather, it suggests that, in a biologically meaningful subset, the gastrointestinal interface may help shape the disease propagation rather than being simply a reflection [3, 86, 88, 91].

Recent work further indicates that α-syn should not be viewed only as a neuronal aggregation-prone protein, but also as an immune-active molecule with systemic consequences [6, 93]. α-Syn is expressed outside the CNS and has been implicated in hematopoietic regulation, immune-cell function, inflammatory activation and age-related immune remodeling [6, 93–95]. This is particularly relevant to the GNU because mucosal macrophages, dendritic cells, enteric glia and epithelial stress pathways are positioned to encounter microbial products, barrier-derived inflammatory cues and α-syn-associated pathology within the local interface [94, 95]. Pathological α-syn may further induce activation of NLRP3 inflammasome, thereby linking proteinopathy to sustained neuroimmune activation [96]. In this context, inflammaging may lower the threshold for the coupling of intestinal α-syn pathology to chronic peripheral and central inflammation [93]. The GNU therefore identifies the gut mucosal interface as a potential site where α-syn-related immune responses, altered mucosal inflammatory activity and gut–brain signalling may interact to influence PD susceptibility and progression [6, 93–96].

Clinical intervention data in PD are broadly consistent with this view. A randomized trial of a four-strain probiotic reported changes in gut microbiota, inflammatory measures and clinical symptoms in PD patients [97]. In parallel, mechanistic reviews have increasingly emphasized the enteroendocrine and GLP-1 signaling as part of the gut-brain axis in PD, rather than treating dysbiosis as a generic trigger. This shift moves the field closer to the GNU by localizing the problem to discrete endocrine-neural interfaces within the mucosa [3, 88]. At the same time, a recent phase 3 trial showed that exenatide administered once weekly failed to demonstrate disease-modifying efficacy in PD [98]. Taken together, these findings support the translational relevance of the GNU, while also indicating that current interventions remain too broad to directly target the GNU.

**Fig. 3:**
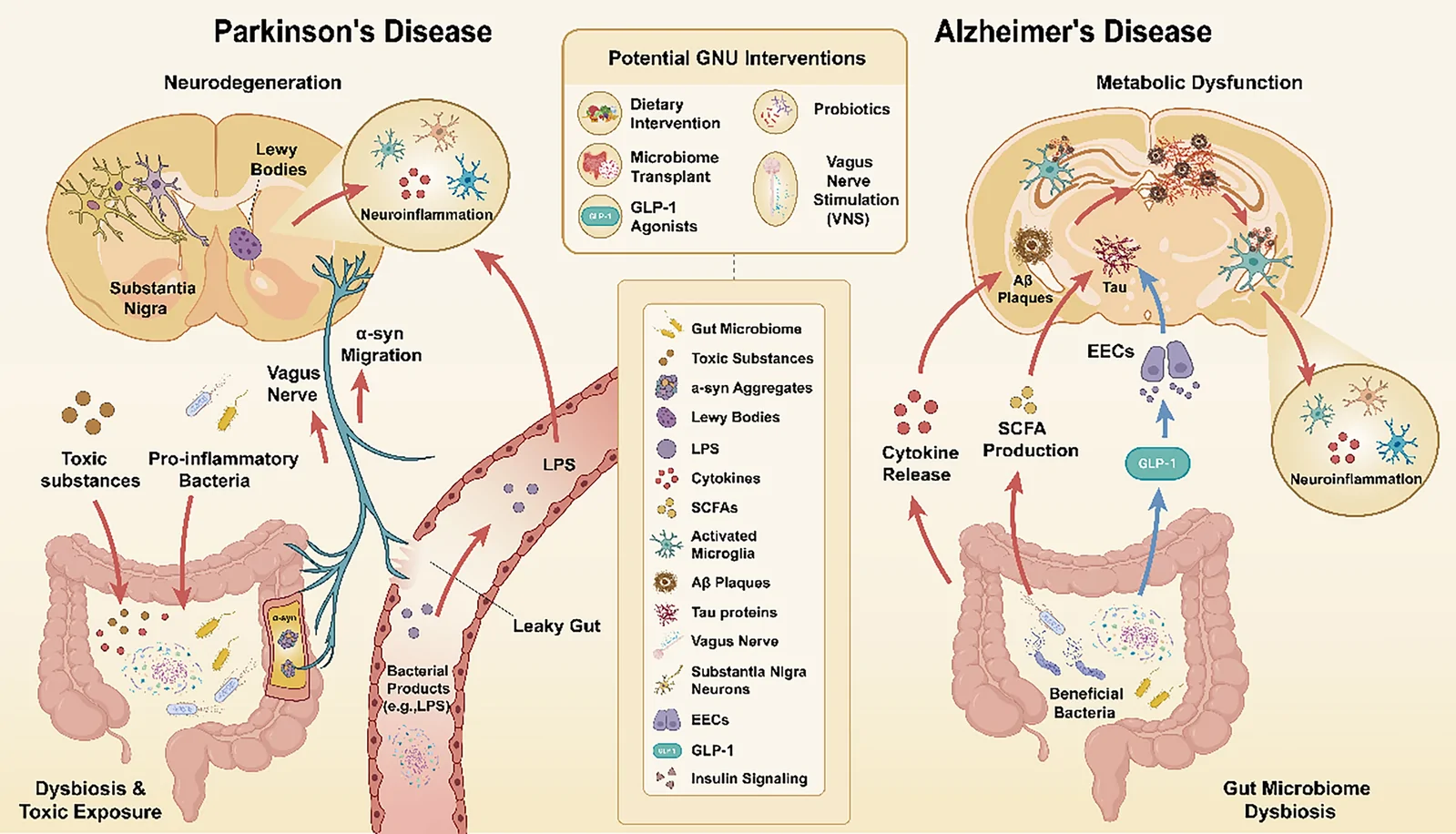
Disease-associated roles of GNU in PD and AD. The figure summarizes how dysfunction of the GNU links peripheral mucosal perturbations to central pathological processes in PD and AD. In PD, dysbiosis, barrier disruption, bacterial products and toxic exposures are associated with intestinal α-syn pathology, vagal/autonomic involvement, neuroinflammation and nigrostriatal degeneration. In AD, the GNU is presented more cautiously as a candidate modulatory interface that may influence inflammatory, metabolic and enteroendocrine gain on established CNS pathology, including Aβ plaques, tau/neurofibrillary pathology, neuroinflammation and brain metabolic dysfunction. Candidate GNU-directed interventions are shown as shared mechanistic categories rather than disease-specific therapies, including luminal input modulation by diet, probiotics or fecal microbiota transplantation, circuit gain modulation by vagal neuromodulation, and neuroepithelial signaling modulation through enteroendocrine pathways such as GLP-1-related signaling. These strategies are depicted as potential approaches for retuning GNU-level input, gain or output, not as established disease-modifying treatments

In AD, the GNU is better viewed as an interface that modulates inflammation and metabolic stress on existing pathology rather than as a universal site of initiation. Human data support an inflammatory link. Fecal calprotectin increases with age and is elevated in amyloid-positive AD, with higher levels associated with greater amyloid burden and poorer verbal memory [99]. Cross-sectional studies have also reported alterations in gut microbial composition in AD patients, which may be related to cognitive symptoms and lifestyle, consistent with a disease-associated shift in mucosal input [100]. In AD mice, germ-free conditions attenuate amyloid and tau pathology, suppress microglial activation, and alter inflammatory and metabolic pathways in the brain. Recolonization with microbiota from AD patients exacerbated pathology and cognitive dysfunction, with polyunsaturated fatty acid-associated neuroinflammation and activation of the C/EBPβ-asparagine endopeptidase axis emerging as one mechanistic route [101]. Recent reviews propose that gut microbiota-driven neuroinflammation accelerates AD [89]. These findings suggest that the GNU may help determine whether microbial and inflammatory signals remain peripheral or are converted into sustained brain-directed inflammatory pressure [89, 99, 101]. Clinical translation remains preliminary. A meta-analysis suggests that probiotics can improve selected cognitive outcomes in mild cognitive impairment and AD, although heterogeneity remains substantial [102]. Likewise, a phase 2b trial of GLP-1 agonist liraglutide in mild to moderate AD patients did not improve the primary endpoint (cerebral glucose metabolic rate), although secondary measures suggested selective benefit [103]. These findings support therapeutic potential, but did not yet provide direct evidence for effective targeting of GNU signaling (Fig. 3).

HD is a genetically defined disease accompanied by prominent peripheral and gastrointestinal features [5, 104]. Early evidence of gut dysbiosis in HD was first reported in a transgenic mouse model, followed by the first clinical study demonstrating altered gut microbial composition in patients with HD and associations with cognitive performance and clinical outcomes [105, 106]. Mutant huntingtin is expressed beyond the CNS, and patients and models show metabolic abnormalities, intestinal dysfunction, weight loss and altered gut microbial profiles. These observations do not imply that HD is initiated by the gut. Rather, they suggest that a genetically driven CNS disease may still be modified by mucosal metabolic and immune states. In HD models, fecal microbiota transplantation can ameliorate dysbiosis and improve selected cognitive outcomes, supporting the concept that the microbiota–mucosa–brain communication can influence disease expression even when the primary genetic lesion is fixed [107]. Within the GNU framework, HD illustrates a modulatory rather than an initiating role of the gut interface. Changes in mucosal sensing, barrier integrity, microbial-derived metabolites and immune signalling states may modify disease trajectory and symptom expression, while mutant huntingtin remains the fundamental driver of disease initiation.

In depression, GNU plays a role by converting microbial, immune and metabolic states into mood-relevant neural activity. Reviews of the microbiota-gut-brain axis in depression converge on three linked mechanisms. First, immune signaling alters the neuroinflammatory tone and behavioural state [90, 92]. Second, disturbance of tryptophan metabolism shifts the balance among serotonin, kynurenine and indole pathways, thereby reshaping the biochemical substrate of affective regulation [108]. Third, microbial composition and the metabolomic state influence antidepressant response, including the efficacy of selective serotonin reuptake inhibitors [109].

Intervention data are broadly consistent with this interpretation. A meta-analysis in clinically diagnosed populations indicates that probiotics can reduce depressive symptoms, whereas prebiotics do not show a comparable effect [108]. Another meta-analysis further suggests that these effects are strain-specific rather than broad [110]. This is consistent with the GNU logic: if the relevant interface is local and mechanistically constrained, broad microbiota manipulation should not be expected to produce uniform outcomes [108, 110]. Vagus nerve stimulation provides a complementary line of evidence. In treatment-resistant depression, vagus nerve stimulation modulates inflammatory measures in the plasma, consistent with a model in which gut-brain communication is shaped by circuit-level as well as biochemical modulation [111]. Depression therefore may be better framed as a disorder in which the mucosal integration interface biases the central vulnerability, symptom persistence and treatment responsiveness through immune, metabolic and neural routes [90, 92, 109, 111, 112].

In conclusion, the GNU is unlikely a universal site of disease initiation. In PD, it may participate early in pathogenesis in a subset of patients [3, 86, 88, 91]. In AD, it is more likely to regulate inflammation and metabolic stress on existing pathology [89, 99, 101, 103]. In depression, it may shape the mood-relevant signaling and treatment response without serving as a singular disease origin [90, 92, 108, 110]. Across these disorders, the common feature is not a shared disease-specific mechanism, but the involvement of a specialized mucosal interface that integrates luminal and tissue-derived signals and converts them into coordinated neural, endocrine and immune responses with potential effects on the CNS [88, 90–92]. Defining this interface provides a more precise framework for experimentally investigating causal mechanisms within the gut–brain axis. Future studies should therefore move beyond describing isolated changes in microbial composition, cytokines or metabolites and instead determine how changes in GNU cellular states, neural pathways and immune responses interact to influence disease-related outcomes.

## Targeting the GNU

The rationale for targeting the GNU does not rely on considering it as a universal initiating cause of disease. Rather, it is based on its role as a modifiable peripheral interface where microbial, nutritional and inflammatory inputs can be regulated, and where epithelial, neural and immune signalling responses may be adjusted [8, 23, 28, 113]. This implies that the intervention should retune how the intestinal interface weights and transmits information, instead of silencing a single pathway [8, 28, 114]. There are three translational routes for intervention as suggested by current literature: (1) targeting the luminal input through microbial or nutritional manipulation, (2) modifying afferent and efferent neural activities through neuromodulation, and (3) targeting druggable signaling nodes located within or immediately adjacent to neuroepithelial processing [8, 114, 115].

The microbiota-based interventions primarily act at the level of input composition by reshaping the luminal chemistry that the GNU detects and encodes. In PD, a meta-analysis shows that probiotic supplementation improves gastrointestinal motility and selected motor and non-motor outcomes, although the effect size and consistency remain variable across studies [116]. A recent meta-analysis in mild cognitive impairment and AD reported improvement in global cognition and selected cognitive domains after probiotic supplementation [102]. An umbrella review further concluded that the probiotic-associated cognitive benefits are supported by moderate- to high-quality evidence across several cognitive outcomes, while also emphasizing heterogeneity in strains, dose and duration [117]. Regarding mood outcomes, evidence is likewise suggestive rather than definitive. A meta-analysis in clinically diagnosed psychiatric populations indicates that probiotics can reduce depressive symptoms, whereas prebiotics do not show a comparable effect [108]. A second review further emphasizes the variability of effects among strains and the need to clarify mechanisms before generalization [110]. These intervention data are mechanistically plausible because enteroendocrine and enterochromaffin cells function as gatekeepers of microbiome–gut–brain communication and translate luminal chemistry into neural and endocrine responses [7, 28, 46]. Further support for this concept comes from the regulation of serotonin signalling by host–microbe interactions. Commensal bacteria can enhance colonic serotonin biosynthesis, and microbial-derived SCFAs further stimulate serotonin production by enterochromaffin cells [83, 118]. These findings suggest that modifying the intestinal environment, including microbial composition and metabolite availability, may alter GNU signalling and gut–brain communication through epithelial sensory pathways. Such approaches may provide a means to modulate GNU function without directly disrupting neural circuits or inducing broad systemic immunosuppression.

Neuromodulation acts on the GNU by altering the afferent-efferent gain and the routing of mucosal information into central circuits. The vagus nerve remains the most tractable conduit because it links gut sensory traffic to brainstem circuits while returning descending control to the intestine [8, 115]. Clinical evidence indicates that this route is modifiable. A meta-analysis of randomized controlled trials found that transcutaneous auricular vagus nerve stimulation improved depression scores and showed a response profile broadly comparable to antidepressant treatment, although the certainty of evidence remained low to very low [115]. A recent experimental study has identified a body–brain axis. Through this axis, pro-inflammatory and anti-inflammatory cytokines communicate with distinct vagal neuronal populations to inform the brain, and the brain in turn regulates systemic inflammatory response [85]. However, vagal nerve stimulation does not directly normalize GNU function in every disease context. From the translation perspective, neuromodulation is feasible because the GNU is embedded within afferent-efferent loops that can be externally modulated. The current limitation is the low resolution: most interventions alter system-level gain rather than modulating specific neuroepithelial microcircuits.

Pharmacological intervention more directly targets the signaling nodes of the GNU. Several targets are already apparent. Enterochromaffin cells couple chemical stimuli to sensory pathways through serotonin release [46]. Neuropod-forming enteroendocrine cells establish direct synaptic communication with vagal neurons and transmit nutrient information within milliseconds [7]. These observations identify serotonin and glutamatergic neuroepithelial transmission as well as enteroendocrine hormone pathways as tractable signaling layers. In an MPTP mouse model of PD, the 5-HT4 receptor agonist prucalopride improved motor dysfunction and attenuated intestinal barrier impairment [103]. In a phase 2b trial in mild-to-moderate AD patients, the GLP-1 agonist liraglutide was generally safe and well tolerated, did not improve the primary endpoint of cerebral glucose metabolism, but showed benefits on a secondary executive measure [118]. These findings argue against the simplistic expectation that a single enteroendocrine pathway will rescue complex CNS disease. Instead, they suggest the feasibility of GNU-targeting therapy when framed to selectively retune a peripheral integrative interface. The most potential candidates are therefore unlikely to be putative master switches, but disease-specific combinations that modify luminal input, vagal gain and neuroepithelial signaling in parallel.

## Conclusion

The value of the GNU lies not in the indication of intestinal complexity, but in the advancement in gut–brain research [80, 113]. Defined as a mucosal interface where epithelial, neural, glial and immune signals converge, the GNU offers a framework for identifying how peripheral events are translated into biologically meaningful neural responses [67, 80, 119]. This is particularly relevant to neurodegenerative diseases. In PD, gastrointestinal dysfunction, microbial alterations and enteric α-syn pathology often precede motor symptoms, yet the mechanisms linking intestinal disturbances to progressive neural injury remain unclear [86, 91]. Similarly, in AD, dysbiosis, barrier dysfunction and chronic inflammation are associated with disease progression, but causal pathways are poorly defined [120, 121]. The GNU provides a mesoscopic scale for investigating how specific epithelial–neuronal–immune interactions encode microbial and metabolic signals that influence brain function [67, 119].

Beyond neurodegeneration, gut–brain interactions are implicated in mood disorders, stress responses and visceral sensitivity [80, 113]. Dietary factors can reshape microbial metabolism and alter the production of microbial-derived metabolites that influence host physiology [122], while neuropod cells rapidly transmit nutrient and microbial information to vagal pathways through specialized epithelial–neural communication mechanisms, including neurotransmitter-mediated signalling [67, 81]. These findings support the concept that the gut actively encodes environmental information through specialized epithelial-neuronal circuits rather than acting as a passive source of systemic signals.

Emerging anatomical and cellular evidence further supports this framework. Single-cell analysis has revealed different cell compositions across different regions of the intestine as well as the complexity of epithelial subtypes [27]. In addition, enteric glia exhibit diverse roles in barrier maintenance, neuronal support and immune regulation [36, 37]. These findings indicate that the gut–brain communication depends on localized cellular networks whose function varies across space and physiological states [119].

Future studies should prioritize high-resolution spatial mapping and causal interrogation of defined mucosal circuits. GNU defines a mucosal unit in which epithelial sensing, neural relay, glial regulation and immune modulation can be mapped, and may be perturbed and linked to disease-relevant outcomes. Future studies should therefore test specific mucosal signaling sequences: the sensing cell, the adjacent responder, the mediator, the timescale, the route and the downstream neural or immune consequence. GNU is an experimentally tractable framework for understanding neurodegeneration, rather than being a simple indication of intestinal complexity [67, 81, 119, 123].

## Data Availability

No datasets were generated or analysed during the current study.

## Abbreviations

AD: Alzheimer's disease
CNS: Central nervous system
CCK: Cholecystokinin
ENS: Enteric nervous system
GLP-1: Glucagon-like peptide 1
GNU: Gut neuroepithelial unit
LPS: Lipopolysaccharide
NEBs: Neuroepithelial bodies
PD: Parkinson's disease
PYY: Peptide YY
SCFA: Short-chain fatty acid
α-syn: α-Synuclein
5-HT: 5-Hydroxytryptamine
